# Supplementary figures and images for: A cross-sectional study of the nasal and fecal microbiota of sows from different health status within six commercial swine farms
Source: PeerJ. 2021 Sep 17;9:e12120. doi: 10.7717/peerj.12120 (PMC8451438; doi:10.7717/peerj.12120)

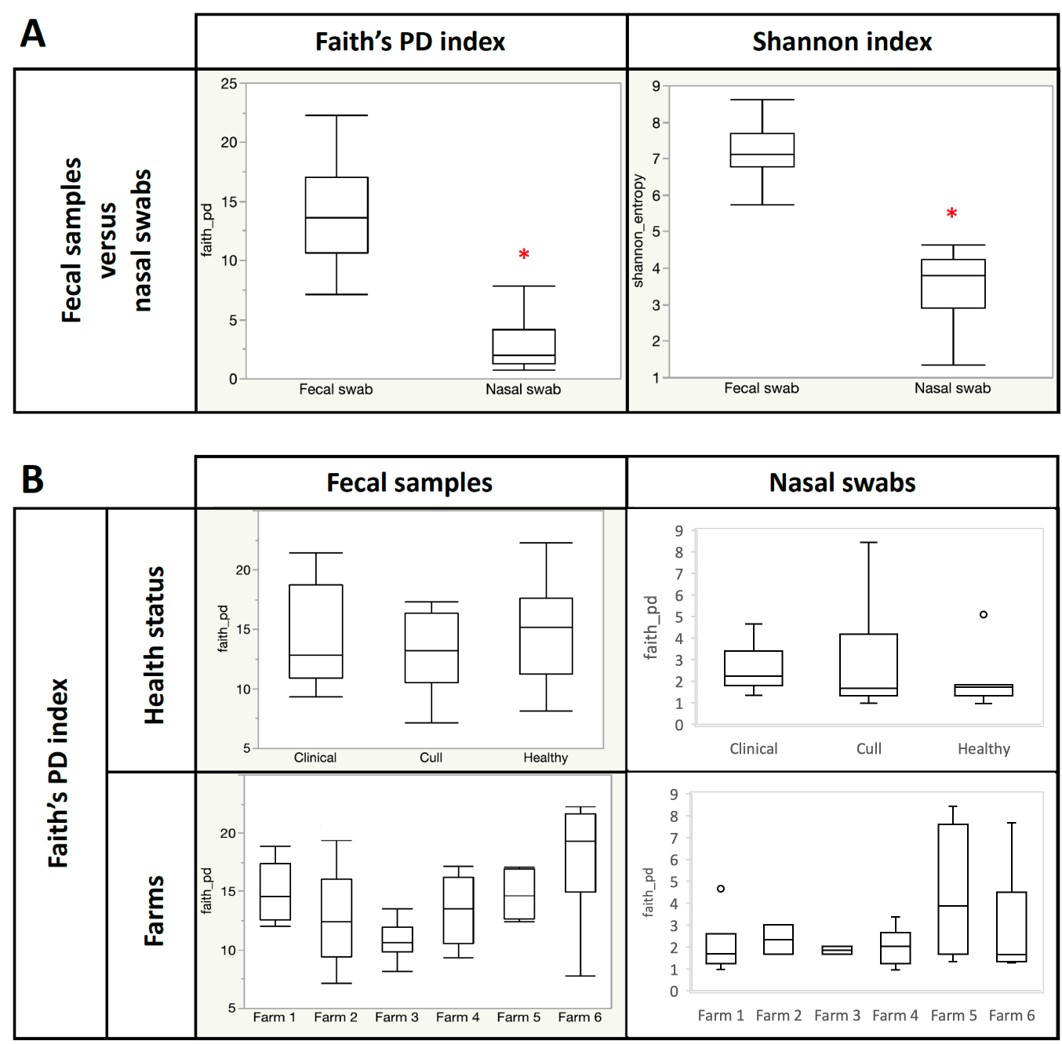

Supplement: Supplemental Information 7 — Microbial diversity by a) sample type and b) farm and health status. In A, all nasal and fecal samples were rarefied at 500 reads. In B, fecal samples were rarefied at 5600 reads and nasal samples were rarefied at 500 reads. Median and quartiles are shown in the box and whiskers plots. N = 36 fecal and 19 nasal swabs. *p <0.001 [file peerj-09-12120-s007.png]

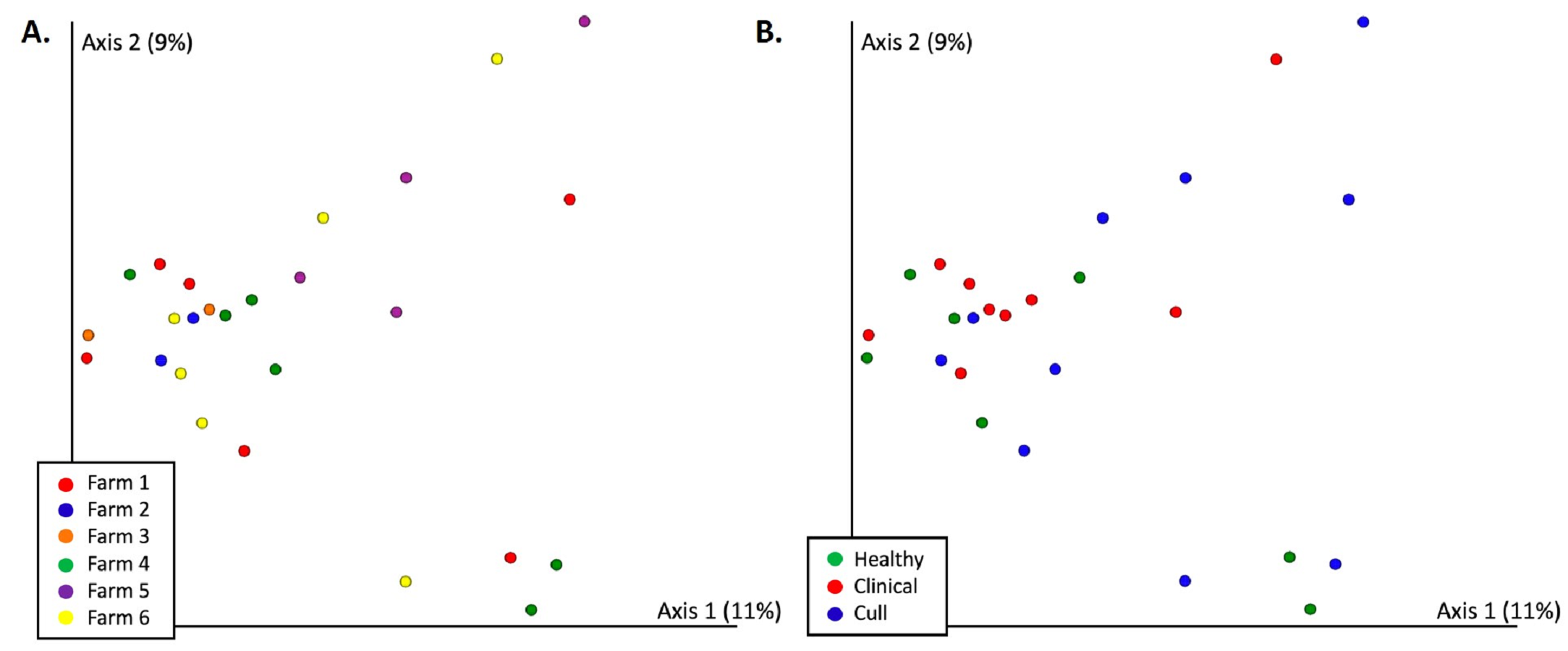

Supplement: Supplemental Information 8 [file peerj-09-12120-s008.png]

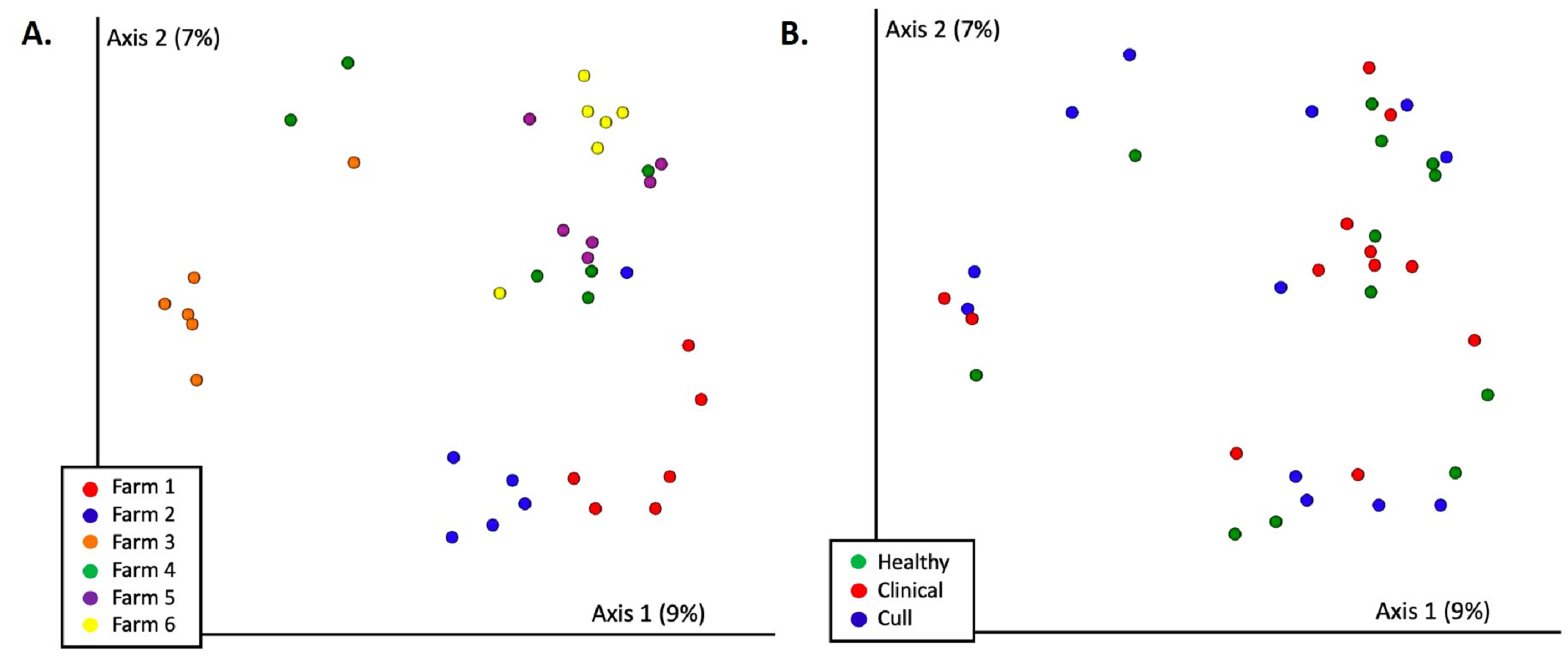

Supplement: Supplemental Information 9 [file peerj-09-12120-s009.png]
